# Use of a Pediatric Admission Booklet Significantly Improves the Comprehensiveness of Admission Documentation: A Quality Improvement Project

**DOI:** 10.1097/pq9.0000000000000247

**Published:** 2020-01-31

**Authors:** Andrew Beverstock, Carianne Lewis, David Bruce, James Barnes, Alison Kelly

**Affiliations:** From the * Department of Pediatric Orthopaedics, Bristol Royal Hospital for Children, Bristol, UK; † Department of Pediatric Rheumatology, Bristol Royal Hospital for Children, Bristol, UK

## Abstract

At present, doctors in some tertiary pediatric hospitals across the United Kingdom record admission on blank continuation sheets rather than using a specific admission document. Previous evidence from adult medicine shows that using admission booklets to prompt the admitting doctor improves the thoroughness of admission documentation, but no work has evaluated this in pediatrics.

**Methods:**

Documentation standards for pediatric admissions were created using national standards. We performed a baseline audit of admissions documented on blank continuation sheets. We included 120 patient admissions across pediatric medicine, pediatric surgery, and pediatric orthopedics (40 from each specialty). We introduced an admission booklet for each specialty, which contained prompts for documenting each aspect of the medical history. We then repeated the audit of 120 additional admissions documented on these booklets.

**Results:**

On average, across all 3 specialties, there was a 33% increase in the inclusion of items in the documented history after the introduction of an admission booklet. In particular, documentation of medication history improved from 46% to 99%, and documentation of allergies improved from 47% to 93%. These improvements were statistically significant.

**Conclusion:**

We recommend the use of a pediatric admission booklet as a simple and effective way to improve medical record documentation. The use of these booklets was associated with an increase in the thoroughness of the documentation. As NHS hospitals transition to electronic medical records, they should make use of admission templates that retain the advantages of these paper booklets.

## Abstract 2

Supplemental Digital Content is available in the text.

## BACKGROUND

Admitting patients is a vital role of junior doctors (a UK term for any non-consultant doctor) within all hospitals. Junior doctors must take an accurate and comprehensive history to allow the construction of a differential diagnosis and to begin treatment. These trainees must document the differential diagnosis in the medical record as part of a succinct and thorough summary of the patient’s history. Documentation of this initial admission history is extremely important for safe patient care: when a doctor from another team needs to review a patient urgently, the admission note is often one of the first documents that they review. Doctors use it when writing discharge letters to enable handover of information to the primary care practitioner. Therefore, the information contained in the admission documentation must be complete and correct. Time pressures on junior doctors can often lead to poor documentation, with significant omissions of vital information.^1^ This poor documentation decreases patient safety by impairing the handover of information between teams and creates re-work for doctors who must re-gather basic information later.

No national standardized electronic medical record system (EMR) exists in England. In 2016, the British government aimed for the NHS to be “paper-free at the point of care” by 2020,^2^ but by 2019, this target had slipped to 2024,^3^ highlighting the scope of the challenge. The UK government imposed EMR systems on hospitals in a top-down fashion, unlike in the United States, where hospitals could choose a record that suited them.^4^ Scotland successfully implemented the TrakCare EMR (InterSystems, Cambridge, Mass.) by allowing clinicians to lead the implementation with minimal government involvement.^5^ In the United States, hospitals were incentivized to adopt EMRs by reducing Medicare and Medicaid reimbursements for those without an EMR, but no such incentive exists in the United Kingdom.^6^ Across the rest of Europe, there is a significant variation in the rates of adoption of EMRs, ranging from 100% of primary care offices in Denmark and Germany to <30% in Switzerland.^7^ Universal adoption has been hindered by fears about privacy breaches, cybersecurity risks, and legal restrictions across EU member states.^8^

Our hospital uses the commercially-available ICE Electronic Health Record (ICE Health Systems, Henderson, Nev.) to order and display investigation results, but doctors write all other documentation on paper that is then scanned into the Evolve medical record system (Kainos, London) after discharge. In our hospital, pediatric admissions were previously documented on blank continuation sheets before this project. We conducted a telephone survey of 5 tertiary pediatric hospitals of similar size to ours in the United Kingdom to try to discover if a similar project had been completed elsewhere. This survey revealed that using a specific admission booklet for documentation of admissions is not routine: 4 used a system similar to our hospital, and only 1 had a completely digital record with an admission template. None had ever used an admission booklet. The effectiveness of admission booklets has been well-established in adult medicine,^9^ adult surgery,^10–12^ and mental health.^13^ Previous work in adult medicine demonstrated that using a specific admission booklet leads to more thorough documentation of social history, examination findings,^14^ and investigation results^15^ without significantly increasing the time to admit patients.^9^

To our knowledge, no similar work has evaluated admission booklets in a pediatric setting. There are key differences between adult and pediatric history taking. Knowing the identity of the adult who has brought a child to hospital is vital because if there are safeguarding issues, it is important to determine if the given history changes over time. Likewise, immunization history is rarely asked about in adult medicine but should be routinely asked in pediatrics because this can alter the differential diagnosis for a child’s presentation and highlight the need for parental education. We audited documentation in our hospital and found that admission documentation was often lacking key information. To address this deficiency, we devised and introduced 3 pediatric admission booklets (1 each for medicine, surgery, and orthopedics) and re-audited admission documentation. We aimed to improve compliance with a set of admission documentation standards from a baseline average of 59% to above 90% after the introduction of the admission booklets.

## METHODS

### Setting/Context

We performed an initial audit at the Bristol Royal Hospital for Children, a tertiary teaching hospital providing regional coverage for the South West of England. The Bristol Royal Hospital for Children provides acute pediatric care for the population of Bristol (535,000 people) and provides specialized pediatric services for a catchment area of 6,000,000 people. The hospital has 166 beds and provides all general pediatric and specialty services, except cardiac transplantation. There are 32,000 emergency department visits per year.^16^ Admission numbers vary depending on the time of year, but typically the hospital admits around 20 pediatric medical patients, 10 pediatric surgical patients, and 5 pediatric orthopedic patients per day. Patients are seen initially by a junior doctor, who writes the admission notes, before being reviewed by a senior consultant on a “post-take ward round.”

### Standard-setting

We created a set of standards using local and national guidelines from the Royal College of Physicians,^17^ which sets standards for medical admission documentation. These guidelines established a set of standards for the information admitting physicians should document in the patient’s medical record upon admission to the hospital. These guidelines are shown in summary form (Table 1).

**Table 1.**
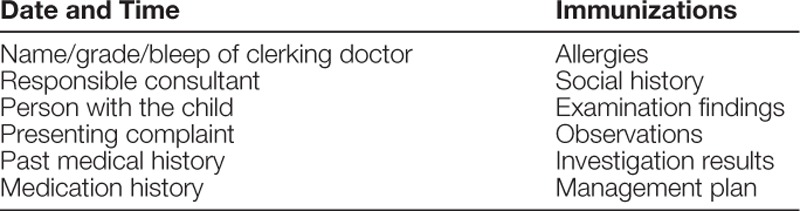
Standards for Patient Clerking Documentation

Our guidelines matched with the standards for digital record keeping jointly developed by the Royal College of Paediatrics and Child Health and the Professional Record Standards Body,^18^ which add pediatric-specific recommendations for information to record.

### Baseline Audit

The admission notes from pediatric medicine, pediatric surgery, and pediatric orthopedics were accessed using the electronic patient record, which contains scanned copies of all paperwork. We identified and reviewed the medical records of 40 surgical, 40 orthopedic, and 40 medical patients in October 2018. We felt this would be a sufficient sample size to detect any significant difference in the thoroughness of the documentation. We selected cases in admission order starting on October first and excluded any cases where the admission paperwork was unavailable on the Evolve system >2 weeks after discharge.

A team of junior doctors reviewed the admission documentation and determined whether it met the standard. Due to the lack of national standards governing information quality beyond inclusion and because it was impossible to standardize judgment of quality between observers, we looked only at whether information about the standard was included or not. We calculated the percentage compliance with each of the admission documentation standards both for the individual specialties and averaged across all 3 specialties. We used the absolute increase in percentage inclusion to measure improvement and allow easy comparison between standards and specialties, as the audit revealed significant variation in the baseline inclusion of each standard.

### Intervention Design

Following this initial audit, we developed an admission booklet for each specialty (see Supplemental Digital Content, available at http://links.lww.com/PQ9/A153). This document was a preprinted paper booklet that contained headings to prompt junior doctors to document aspects of the medical history. The booklet was available in the emergency department, where junior doctors see new admissions. Unlike blank continuation sheets, which have no heading, our booklets were color-coded and were labeled in the margin as “Admission Booklet” to make them easy to identify in the paper medical notes. The color ran to the edge of the paper to make it easy to identify.

We reviewed the proposed admission booklets with the clinical teams, received feedback for how to improve them, and completed revisions to the booklets based on this feedback. After approval for use by the University Hospitals Bristol leadership team and clinical documentation committee, we instructed junior doctors and nurses in their use: project team members attended several junior doctor handover meetings and explained the booklets’ content and purpose. We then introduced them into routine use in March 2019.

### Re-audit

We then identified patients whose admissions were documented using the booklet from patient lists and a review of the medical records. We analyzed the first 40 patients using the booklet for each specialty. We then completed a repeat audit using the same standards as the baseline audit. Forty patient admissions from each specialty (medicine, surgery, and orthopedics) were reviewed and compared with the baseline audit.

### Statistical Analysis

Statistical significance for any improvement in documentation across all standards was determined using a Mann-Whitney *U* test. A binominal calculation was used to determine whether the change for each standard was significant. A *P*-value of <0.05 was considered significant.

## RESULTS

**Fig. 1.**
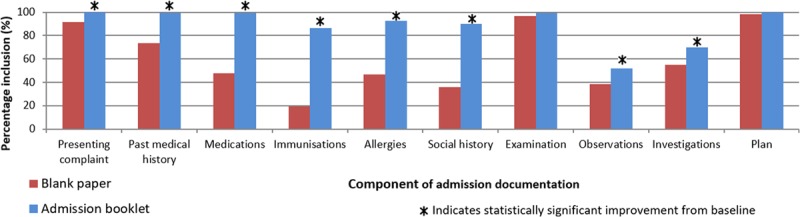
Documentation of medical history on blank continuation sheets compared with admission booklets.

The baseline audit revealed significant omissions from the admission documentation (Fig. 1). Junior doctors rarely documented certain key non-medical information, most notably the source of referral (23% inclusion), site of assessment (10%), responsible consultant (8%), and the person accompanying the child (24%). Medications (48%), allergies (47%), social history (36%), and investigations (55%) were also frequently omitted from patient notes written on blank continuation sheets. Documentation of all standards improved with the introduction of the booklets in all specialties. There was a significant improvement in the thoroughness of admission documentation using booklets across all pediatric specialties (Table 2).

**Table 2.**
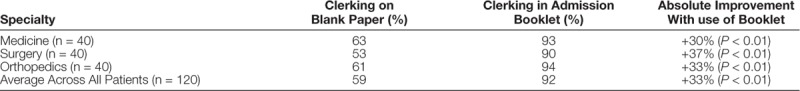
Average Results from All Standards Across All 3 Specialties for Clerkings on Blank Paper Compared with Clerkings in Admission Booklets

On average, across all 3 specialties, there was a 33% absolute improvement in the inclusion of information with the booklets (*P* < 0.01). On the blank paper sheets, no standard reached 100% inclusion. After the introduction of the booklets, 4 standards reached 100% inclusion, and 12 of 17 standards reached >90% inclusion compared with only 5 of 17 standards on blank continuation sheets (Fig. 1).

Documentation of medications improved by 51% (*P* < 0.01), allergies by 46% (*P* < 0.01), and social history by 54% (*P* < 0.01). Non-medical information was also more comprehensively documented in the booklets: the consultant (the most senior doctor) responsible for the patient was documented only 8% of the time on blank continuation sheets, but doctors recorded it in 88% of documentations using the booklets (*P* < 0.01). Recording of the identity of the adult with the child increased from 24% to 68% (*P* < 0.01).

Documentation of the examination findings (3% improvement, *P* = 0.30) and management plan (2% improvement, *P* = 0.15) improved less than the other standards, but these were both well-documented even on blank continuation sheets. Documentation of observations was poor in the baseline audit and improved little even with a dedicated observation section in the booklet (an increase from 38% to 52%, *P* < 0.01).

## DISCUSSION

The use of an admission booklet is a highly effective way of improving the comprehensiveness of admission documentation in all pediatric specialties. There were several advantages to using booklets compared with the previous system of using blank continuation paper sheets. The color-coded booklets are easily identified in the patient notes. In contrast, the continuation sheets can more easily be lost in a thick stack of patient notes. The booklets are stapled together securely; thus, the admission note remains as a single document, preventing it from becoming scattered throughout a large set of notes as frequently happens with loose-leaf paper.

The introduction of a tick-box to indicate a lack of regular medications and allergies meant that doctors could easily document “negative findings” in the patient’s history. On the blank paper, documentation included a medication history 48% of the time. For the remaining patients, it was impossible to tell whether they were not receiving medications or whether the admitting doctor failed to ask about the patient’s medication history. Junior doctors using the admission booklets documented the medication history 99% of the time, making medication reconciliation easier.

Although documentation of all standards improved, there were still significant omissions in several key areas of the medical history, particularly observations (52%) and investigative studies (70%). In our booklet, there was space for the documentation of laboratory studies and test results. However, these results are often only available after the initial admission documentation. While the booklets did improve documentation of test results compared with blank continuation sheets, further staff training would be an effective method for additional improvement by emphasizing the importance of effective documentation.^19^

Junior doctors recorded every aspect more comprehensively in the booklets than on the paper forms. The aspects of the medical history that were well-documented initially (presenting complaint, examination findings, and the management plan) continued to be thoroughly documented in the booklets. Creating a standardized place in the admission note for this information to be documented allows this key information to be easily referenced during the patient admission, and makes it easy for nursing staff to find the management plan in the notes.

Junior doctors are often resistant to using new paperwork. Encouraging the use of an admission booklet proved challenging initially as some doctors were worried about an increased workload as a result of the booklet. To alleviate this concern, we involved junior doctors in the creation of the booklet to make sure that it was easy to complete. We involved all staff in the process to ensure a booklet that was fit for purpose. Pharmacists, ward clerks, nurses, and hospital coders were all consulted during the creation of the booklet. Gaining this buy-in during a project is vital to ensuring that the change is sustainable: one-third of quality improvement projects are not sustained 1 year after implementation.^20^ Planning sustainability at the design stage and addressing it early in project implementation is the only way to sustain change effectively.^21,22^ We did this by ensuring that we consulted senior management about the project and that both management and front-line users were consulted about the project before its implementation. We also sought feedback after implementation.

This project highlights the advantage of cross-specialty working: pediatricians can adapt simple ideas that are ubiquitous in adult medicine into their practice. Pediatricians should look to adult medicine for quality improvement ideas, and vice versa, as different specialties can learn from each other. Anecdotally, after we introduced the booklets, 1 of our trainees reported a positive experience of using a similar pediatric booklet at a smaller UK district general hospital demonstrating how smaller units can lead specialist centers in the efficient delivery of safe patient care and how, as pediatricians, we are all experiencing the same challenges.

### Limitations

We examined whether doctors included the information without assessing the quality of that documented history. We looked at whether the documentation was complete. We would need to complete a separate project to tell if the information was of a higher quality when documented in a booklet. We used a list of standards and discussed in advance what information would be acceptable to meet each standard to reduce variability in data collection. Additionally, this project did not look at patient outcomes, and thus we could not measure whether the booklets improved patient safety. Finally, our project is only a short-term solution: this intervention will not be required once the NHS introduces an EMR, and thus our intervention is naturally time-limited in its utility.

We did not achieve 100% compliance with all standards. Possible reasons for this include time pressure for doctors on busy shifts making it difficult to take the time to document comprehensively or a lack of understanding of the importance of thorough documentation. In the future, additional training for junior doctors may help to alleviate these issues.

We did not record the identity of the doctors documenting the admissions to allow staff anonymity. Junior doctors rotate through different hospital departments over the year, and therefore, some of the variation in results came from sampling a different set of doctors. We completed this project in a single training year to try to reduce this variability, but the time-frame from the initial audit through to booklet implementation made it impossible to ensure absolute continuity in admitting staff.

## CONCLUSIONS

Specialty-specific admission booklets are an easily implemented and effective method of improving documentation of pediatric admissions in the medical record. The presence of an on-paper prompt for junior doctors encourages them to ask about typically neglected parts of the medical history, such as social history, and ensures that they record these elements during the admission process. The use of tick boxes to indicate the absence of regular medications allows the differentiation of patients who do not take any medications from those cases where the admitting doctor did not take a thorough medication history. This addition is an easy intervention to the quality of the medical record.

Any hospital without an EMR that uses blank continuation sheets to document patient admissions should consider implementing booklets like ours. In the future, as hospitals move toward “paperless notes,” these booklets should be replaced by admission templates on the EMR. An admission template maintains the advantages of having prompts to remind doctors to document histories thoroughly and ensures that information is logically organized in the electronic record.

## DISCLOSURE

The authors have no financial interest to declare in relation to the content of this article.

## Supplementary Material


