# Supplementary material for: Use of a Pediatric Admission Booklet Significantly Improves the Comprehensiveness of Admission Documentation: A Quality Improvement Project
Source: Pediatr Qual Saf. 2020 Jan 31;5(1):e247. doi: 10.1097/pq9.0000000000000247 (PMC7056295; doi:10.1097/pq9.0000000000000247)
Supplement: Supplementary file 1 [file pqs-5-e247-s001.docx]

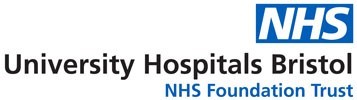


**Paediatric Medical Admission Booklet**


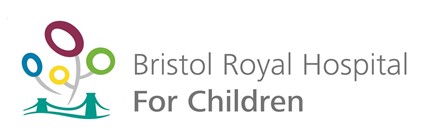


PATIENT DETAILS (attach sticker) Forename

Surname

DOB

T number

Gender

Date: Time: Name/grade/bleep:

Admitting specialty:

Admitting consultant:

Source of referral: Location seen: Person accompanying child:

Weight: kg

**Safeguarding concerns? Yes / No**

MEDICAL PAEDIATRIC ADMISSION BOOKLET

**Presenting complaint**

**History of presenting complaint**

*History taken from:*

Evolve Doc Type: Inpatient Clinical Notes 1 **THQ 372**


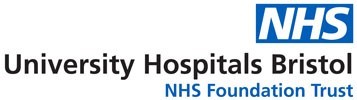

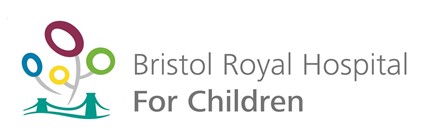


**Past medical/surgical history**

PATIENT DETAILS (attach sticker) Forename

Surname

DOB

T number

Gender


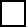


**ALLERGIES** No known drug allergies

Signature

**Medications**

No regular medications


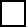


**MEDICATIONS**

**DRUG DOSE ROUTE FREQUENCY**

MEDICAL PAEDIATRIC ADMISSION BOOKLET

Immunizations up-to-date? Yes/No If not, why?

2


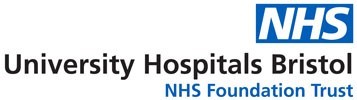


**Social and Family History**


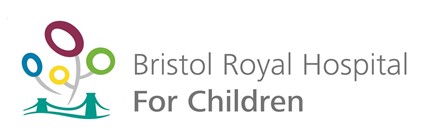


*Social worker involved? Y/N*


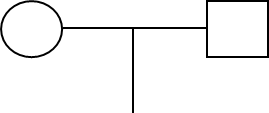


PATIENT DETAILS (attach sticker) Forename

Surname

DOB

T number

Gender

**Admission observations**

*HR*

*Temp*

*BP*

*O2 sats % on*

**Examination**

*General*

*Respiratory ENT*

*Abdominal*


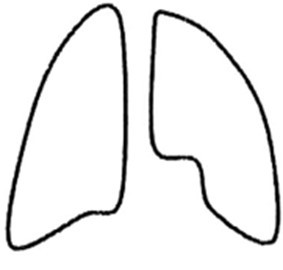

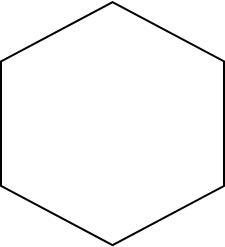


*Cardiovascular*

*Neurological Musculoskeletal*

*Development Skin*

*RR*

*Glucose*

MEDICAL PAEDIATRIC ADMISSION BOOKLET

Evolve Doc Type: Inpatient Clinical Notes 3 **THQ 372**


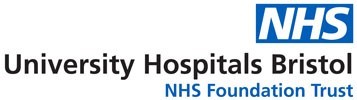

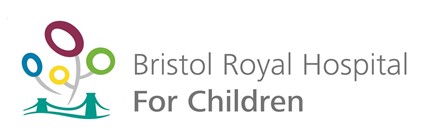


**Impression**

PATIENT DETAILS (attach sticker) Forename

Surname

DOB

T number

Gender

MEDICAL PAEDIATRIC ADMISSION BOOKLET

**Management**

*Consider consulting the BRHC management guidelines on the hospital intranet*

Name Grade Bleep Signature

4

**Post-Take Ward Round**


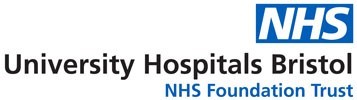

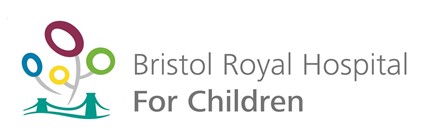


Consultant present on ward round? **Yes / No**

Name of consultant: Date & time:

PATIENT DETAILS (attach sticker) Forename

Surname

DOB

T number

Gender

*Management plan discussed with nurses? Y / N Name of nurse:*

MEDICAL PAEDIATRIC ADMISSION BOOKLET

**Name Grade Bleep Signature**

Evolve Doc Type: Inpatient Clinical Notes 5 **THQ 372**

**Continuation Sheet**

PATIENT DETAILS (attach sticker) Forename

Surname

DOB

T number


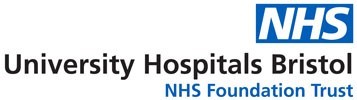


Gender

MEDICAL PAEDIATRIC ADMISSION BOOKLET


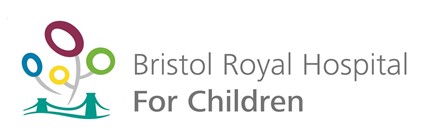


6

**Continuation Sheet**


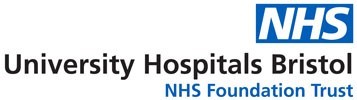


PATIENT DETAILS (attach sticker) Forename

Surname

DOB

T number

Gender

MEDICAL PAEDIATRIC ADMISSION BOOKLET


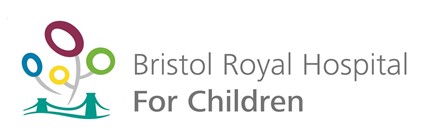


Evolve Doc Type: Inpatient Clinical Notes 7 **THQ 372**

MEDICAL PAEDIATRIC ADMISSION BOOKLET

PATIENT DETAILS (attach sticker) Forename

Surname


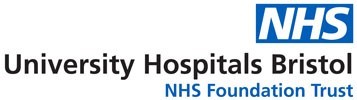


DOB

T number

Gender

**Date:**


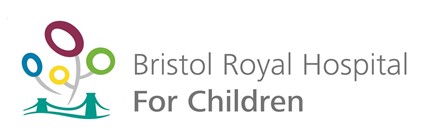


CRP WCC

Hb HCT MCV Plt Neut

Na K Urea Creat

Total bil

ALP ALT Alb

Tot. prot

Globulin

Mg

PO4

CrCa2+

Amylase

ESR

Plasma visc.

*Urinalysis*

Date: Time: :_

MSU sent? Y/N β-hCG positive / negative

pH Nit Leu

Glu Pro

Ket Blood

8
